# Diagnostic performance of the ECG-SMART AI score for detecting angiographically significant coronary artery disease in patients undergoing elective coronary angiography

**DOI:** 10.1371/journal.pdig.0001701

**Published:** 2026-10-08

**Authors:** Dania A. Bani Hani, Jafar A. Alshraideh, Hamza Alduraidi, Akram Saleh, Nathan T. Riek, Karam Daoud, Rui Qi Ji, Samir Saba, Clifton W. Callaway, Salah Al-Zaiti

**Affiliations:** 1 School of Nursing, Middle East University, Amman, Jordan; 2 School of Nursing, University of Jordan, Amman, Jordan; 3 Department of Cardiology, Jordan University Hospital, Amman, Jordan; 4 School of Nursing, University of Rochester, Rochester, New York, United States of America; 5 School of Engineering, Texas Tech University, Lubbock, Texas, United States of America; 6 Department of Electrical & Computer Engineering, University of Toronto, Ontario, Canada; 7 Division of Cardiology, University of Pittsburgh, Pittsburgh, Pennsylvania, United States of America; 8 INTELLICARDIO INC., Pittsburgh, Pennsylvania, United States of America; 9 Department of Emergency Medicine, University of Pittsburgh, Pittsburgh, Pennsylvania, United States of America; Xinjiang Medical University Affiliated First Hospital, CHINA

## Abstract

The 12-lead ECG is central to the evaluation of patients with suspected coronary artery disease (CAD) but has limited diagnostic accuracy for identifying significant obstructive disease prior to elective coronary angiography. We previously developed an artificial intelligence–enhanced ECG (AI-ECG) model for detecting acute coronary occlusion. Whether this model generalizes to identifying angiographically significant CAD in symptomatic patients undergoing elective evaluation remains unknown. In this prospective cohort study, symptomatic outpatients undergoing elective coronary angiography at a tertiary center received standard 12-lead ECGs before the procedure. ECGs were analyzed offline using a previously validated AI-ECG model originally developed for acute coronary occlusion. Patients were categorized as low or intermediate-to-high risk using predefined thresholds. The primary outcome was angiographically significant CAD (≥70% stenosis in a major epicardial vessel or ≥50% stenosis of the left main coronary artery). Among 363 patients (age 58.9 ± 11 years; 55.1% male), 36.1% had angiographically significant CAD. The AI-ECG classified 56% as low risk and 44% as intermediate-to-high risk. After adjustment for conventional clinical characteristics, intermediate-to-high risk remained independently associated with significant CAD (OR 3.12, 95% CI 1.88–5.20; p < 0.001). Model discrimination was good (AUROC 0.79, 95% CI 0.74–0.84), with 60% precision at 80% recall. In summary, the ECG-SMART AI score demonstrated good diagnostic performance for identifying angiographically significant CAD in symptomatic patients undergoing elective coronary angiography. These findings support the generalizability of a previously developed AI-ECG model beyond acute coronary occlusion and suggest that AI-enhanced ECG analysis may complement existing clinical assessment by providing additional noninvasive information to refine pretest probability during elective evaluation.

## Author summary

Coronary artery disease is one of the leading causes of illness and death worldwide. Identifying patients with important narrowing of the heart arteries can be challenging, particularly when symptoms are not acute. The electrocardiogram is inexpensive, widely available, and routinely used in cardiovascular care, but conventional interpretation has limited ability to detect underlying coronary artery narrowing in stable patients. We previously developed an artificial intelligence approach that analyzes information within a standard 12-lead electrocardiogram to identify patients at risk for an acutely blocked coronary artery. In this study, we asked whether the same approach could provide useful information in a very different setting: symptomatic patients undergoing planned coronary angiography rather than emergency evaluation for a heart attack. Among 363 patients, the artificial intelligence score successfully identified patients more likely to have significant coronary artery narrowing and remained informative after accounting for conventional cardiovascular risk factors. These findings suggest that artificial intelligence may uncover subtle electrical patterns associated with coronary disease that extend beyond acute heart attacks. Further studies are needed to determine whether this approach can help clinicians improve noninvasive evaluation and guide decisions about additional testing in broader patient populations.

## Introduction

Coronary artery disease (CAD) remains the leading cause of mortality worldwide, accounting for nearly 9 million deaths annually [1]. The disease is characterized by a prolonged subclinical phase, often progressing silently until patients present with symptomatic or catastrophic events such as myocardial infarction [2]. Accordingly, early detection and accurate risk stratification of clinically significant CAD are essential to prevent irreversible myocardial injury and guide timely intervention.

The 12-lead electrocardiogram (ECG) is a cornerstone of initial risk assessment in patients with suspected acute coronary syndrome (ACS) [3]. However, its diagnostic performance is largely dependent on overt ischemic patterns, such as ST-segment elevation, which are frequently absent in subacute or stable presentations. Subtle or nonspecific ECG changes are common in these populations, and up to two-thirds of occlusive myocardial infarctions may be difficult to identify using conventional ECG interpretation alone [4]. These limitations contribute to diagnostic uncertainty and may delay appropriate management.

Recent advances in artificial intelligence (AI) have enabled the extraction of high-dimensional features from ECG signals, allowing detection of complex and previously unrecognized patterns associated with myocardial ischemia [5]. The ECG-SMART algorithm, developed to identify occlusive myocardial infarction, leverages information across the entire QRST complex and has demonstrated superior performance compared with traditional risk markers and commercial ECG software in acute care settings [6]. These findings suggest that AI-enhanced ECG analysis can improve early risk stratification and clinical decision-making.

Beyond acute care, AI-based approaches have shown promise in detecting and risk stratifying CAD using imaging modalities such as single-photon emission computed tomography, coronary computed tomography angiography, and echocardiography [7]. However, the application of AI-ECG models in stable or subacute populations remains underexplored [8,9]. It is unclear whether the electrical signatures identified during acute ischemia can also reflect the presence of significant, anatomically obstructive CAD in patients undergoing elective evaluation. Addressing this gap is critical to determine the broader clinical utility and generalizability of AI-ECG tools.

### Purpose

The present study aimed to evaluate the diagnostic performance and incremental clinical value of the ECG-SMART AI model for improving estimation of pretest probability of angiographically significant coronary artery disease among symptomatic patients undergoing elective coronary angiography.

## Methods

### Study design and population

This prospective cohort study enrolled consecutive adult patients (≥18 years) undergoing elective coronary angiography at Jordan University Hospital between February and July 2024. Eligible patients were referred for angiography based on clinical suspicion of obstructive CAD. Consecutive sampling was employed to minimize selection bias. Patients presenting for emergent PCI or with confirmed ST-elevation myocardial infarction (STEMI) were excluded, as the study focused on stable and subacute presentations.

The study was conducted in accordance with the Declaration of Helsinki and approved by the Institutional Review Board of the University of Jordan (IRB-10-2023-31960). Because the study involved secondary analysis of clinically indicated ECGs and routinely collected clinical data without alteration of patient management or additional study-specific procedures, a waiver of informed consent was granted.

### ECG acquisition and processing

Patients underwent standard 12-lead ECG acquisition as part of routine pre-procedural clinical evaluation prior to coronary angiography. To facilitate collection of digital waveform data for research analysis, ECGs were acquired using a portable digital electrocardiograph that had been implemented within the clinical workflow of the study unit. No additional ECG recordings were obtained beyond those required for routine patient care. Digital ECGs were stored in XML format for subsequent offline analysis.

Waveform data were processed using a previously validated signal processing pipeline [6,10,11]. Preprocessing included removal of baseline wander, powerline interference, and high-frequency noise, followed by harmonization of sampling rates, amplitude normalization, and lead configuration standardization. ECGs with excessive noise or ectopic beats that precluded reliable median-beat construction were excluded. Representative median beats were generated independently for each lead after beat alignment using automatically detected fiducial points. Fiducial-point detection enabled segmentation of the P wave, QRS complex, and ST-T complex for downstream feature extraction. The engineered feature set comprised 417 predefined descriptors spanning temporal intervals, amplitudes, waveform areas, spatial axes, vectorcardiographic measures, principal component analysis metrics, spectral characteristics, beat-to-beat variability, and repolarization morphology. A complete list of engineered features is provided in the Supplementary Materials, and detailed descriptions of the preprocessing algorithms have been published previously.

### AI-ECG model application

The extracted ECG features were input into a previously developed and externally validated AI model (ECG-SMART) to estimate the probability of coronary occlusion [6]. The model, based on a random forest classifier, was originally trained on 10,346 ECGs from 7,397 patients across multiple U.S. institutions and demonstrated stable performance in external validation cohorts.

Predefined probability thresholds were applied to classify patients into low-, intermediate-, and high-risk categories. For analysis, intermediate and high-risk groups were combined because the very low number of patients (n = 2) classified as high risk in this cohort. Model interpretability was assessed using SHapley Additive exPlanations (SHAP) to identify the most influential ECG features contributing to predictions.

### Clinical data and outcome definition

Clinical data were abstracted from electronic medical records, including demographics (age, sex), cardiovascular risk factors (e.g., hypertension, diabetes, dyslipidemia, smoking status), prior cardiovascular history, presenting symptoms, and available laboratory data. Data abstraction was performed using standardized forms to ensure consistency and completeness.

Following the procedure, finalized coronary angiography reports were obtained and de-identified for analysis. Two independent physician reviewers, blinded to all clinical, ECG, and AI-derived results, systematically reviewed each report to extract the presence, location, and severity of coronary artery stenosis. Coronary lesions were classified by vessel (left main, left anterior descending, left circumflex, and right coronary artery) and degree of luminal narrowing.

The primary outcome was angiographically significant CAD, defined as ≥70% diameter stenosis in a major epicardial coronary artery or ≥50% stenosis in the left main coronary artery, as determined by the interpreting interventional cardiologist, and consistent with established anatomical thresholds for clinically significant obstructive disease and lesions generally considered for coronary revascularization when appropriate [12]. Fractional flow reserve (FFR) and instantaneous wave-free ratio (iFR) measurements were not routinely performed in this cohort because they were reserved for clinically selected cases rather than obtained systematically in all patients undergoing elective coronary angiography. Consequently, physiology-based measurements were not available for use as the primary study endpoint. When multiple lesions were present, the most severe stenosis was used for classification. Coronary angiograms were independently reviewed by two investigators blinded to the AI-ECG results, with excellent inter-rater agreement (Cohen’s κ = 0.96). Any discrepancies were resolved by consensus through adjudication by a third independent reviewer.

### Statistical analysis

All analyses were performed using IBM SPSS Statistics (version 27; IBM Corp., Armonk, NY, USA). Continuous variables are reported as mean ± standard deviation or median (interquartile range [IQR], 25th–75th percentile), as appropriate based on distribution, and categorical variables as counts and percentages. Group comparisons were performed using Student’s *t*-test or Mann–Whitney *U* test for continuous variables and χ² or Fisher’s exact test for categorical variables.

Model calibration of predicted AI-ECG probabilities was evaluated using calibration intercept and calibration slope derived from logistic recalibration analysis. Calibration plots were generated by comparing observed and predicted event rates across deciles of predicted risk. Overall prediction accuracy was assessed using the Brier score.

Next, we categorized AI-ECG scores to previously derived risk categories. Multivariable logistic regression was used to evaluate the independent association between these AI-ECG risk categories (independent variable) and significant CAD (dependent variable). Covariates were selected based on clinical relevance and observed association with the outcome, using a liberal screening threshold of P < 0.10 to reduce the likelihood of excluding potentially important confounders. To balance adjustment for residual confounding against the risk of overfitting, the final model was limited to a parsimonious set of covariates and included age, sex, smoking status, history of CAD, diabetes mellitus, hyperlipidemia, HDL cholesterol, and triglyceride values. The primary purpose of the multivariable logistic regression was to evaluate whether the ECG-SMART AI score remained independently associated with angiographically significant CAD after adjustment for conventional clinical characteristics. The regression analysis was not intended to develop or benchmark a new clinical prediction model.

Model discrimination was assessed using the area under the receiver operating characteristic curve (AUROC) with 95% CIs. Precision–recall performance was additionally evaluated, and precision was reported at a fixed recall of 80% to reflect clinically relevant sensitivity thresholds. The optimal probability threshold for classification was determined using the Youden Index. Goodness-of-fit of the logistic regression model was assessed using the Hosmer–Lemeshow test. All statistical tests were two-sided, and a *p*-value <0.05 was considered statistically significant.

## Results

### Sample characteristics

A total of 363 patients undergoing elective coronary angiography were included. The mean age was 58.9 ± 11 years, and 200 (55.1%) were male. Cardiovascular risk factors were prevalent, including hypertension (62.5%), diabetes mellitus (44.4%), active smoking (35.0%), prior CAD (33.9%), and prior revascularization (43.8%); 65.3% reported a family history of CAD. Chest pressure was the most common presenting symptom (76.9%). Overall, 131 patients (36.1%) had significant coronary artery narrowing (≥70% stenosis or ≥50% left main).

The AI-ECG model classified 204 patients (56%) as low risk and 159 (44%) as intermediate-to-high risk. Baseline characteristics stratified by AI-ECG risk category are shown in Table 1. Patients in the intermediate-to-high risk group were more likely to be male, active smokers, and to have lower platelet counts and HDL cholesterol levels.

**Table 1:** Baseline clinical characteristics and comorbidities, n = 363.

Variable	Total (n = 363)	AI-ECG Score	
		Low Risk n = 204	Moderate-to-High Risk n = 159
**Age (years)**	59 ± 11	58.7 ± 11.2	59.1 ± 10.7
**Male sex**	200 (55%)	87 (43%)	113 (72%) **
**Comorbidities**			
Active Smoking	127 (35%)	55 (27%)	72 (45%) **
Diabetes mellitus	161 (44%)	89 (44%)	72 (45%)
Hypertension	227 (63%)	128 (63%)	99 (62%)
History of high cholesterol	190 (52%)	104 (51%)	86 (54%)
History of Known CAD	123 (34%)	64 (31%)	59 (37%)
Prior PCI	159 (44%)	81 (40%)	78 (49%)
Chest Pressure (symptom)	279 (77%)	152 (75%)	127 (80%)
Family history of CAD	237 (65%)	135 (66%)	102 (64%)
**Laboratory Findings**			
Hemoglobin (g/dL)	13.2 ± 1.5	13.2 ± 1.4	13.2 ± 1.6
Platelet (×10^3^/µL)	253 ± 74	262 ± 71	241 ± 76 *
WBC (×10^3^/µL)	8.5 ± 5.6	8.2 ± 4.8	8.9 ± 6.5
Neutrophils (%)	57 ± 11.3	56 ± 11.3	58 ± 11.1
Lymphocyte (%)	32 ± 13	33 ± 10.5	31 ± 16.3
Monocytes (%)	8.8 ± 7.6	8.5 ± 4.8	9.2 ± 10.1
Total cholesterol (mg/dL)	175 ± 44	179 ± 42	172 ± 47
Triglyceride (mg/dL)	145 ± 104	171 ± 98	181 ± 112
HDL (mg/dL)	44 ± 14	47 ± 16	40 ± 12 **
LDL (mg/dL)	110 ± 38	113 ± 37	108 ± 39
**Angiography Findings**			
Significant CAD	131 (36%)	46 (23%)	85 (53%) **
Multi-vessels Disease	191 (53%)	89 (44%)	102 (64%) **
LAD narrowing	212 (58%)	108 (53%)	104 (66%) *
LCX narrowing	169 (46%)	78 (38%)	91 (57%) **
RCA narrowing	161 (44%)	71 (35%)	90 (57%) **

**: p < 0.001; *: p < 0.05.

### Association with significant coronary narrowing

On univariate analysis (Table 2), intermediate-to-high AI-ECG risk was associated with a higher prevalence of significant coronary narrowing compared with the low-risk group (53% vs 23%; OR 3.95, 95% CI 2.51–6.20; *p* < 0.001). This association remained significant after adjustment for age, sex, smoking status, history of CAD, diabetes mellitus, hyperlipidemia, HDL cholesterol, and triglyceride values (adjusted OR 3.12, 95% CI 1.88–5.20; *p* < 0.001). Model discrimination was good, with an AUROC of 0.792 (95% CI 0.744–0.839) (Fig 1). Precision–recall analysis demonstrated a precision of 60% at 80% recall, consistent with clinically relevant sensitivity thresholds.

**Table 2:** Univariate and multivariate regression model of severe CAD, n = 363.

Variables	Univariate model			Multivariate model		
	OR	95%CI	*P*	OR	95%CI	*P*
Age	**1.03**	**1.01-1.05**	**0.007**	**1.05**	**1.01-1.09**	**0.005**
Male sex	**3.30**	**2.13-5.14**	**<0.001**	**2.86**	**1.58-5.18**	**0.008**
Smoking	**2.08**	**1.33-3.25**	**0.001**	1.63	0.91-2.92	0.099
Hypertension	0.75	0.48-1.17	0.20	─	─	─
Diabetes	**1.87**	**1.21-2.88**	**0.005**	**1.74**	**1.02-2.96**	**0.041**
History of high cholesterol	**1.63**	**1.05-2.52**	**0.028**	1.37	0.82-2.33	0.229
History of CAD	**2.92**	**1.86-4.60**	**<0.001**	**1.96**	**1.16-3.31**	**0.012**
Family history	1.07	0.68-1.68	0.77	─	─	─
WBC (×10^3^/µL)	1.01	0.97-1.04	0.78	─	─	─
Triglyceride (mg/dL)	**1.00**	**1.00-1.01**	**0.05**	1.00	0.99-1.01	0.82
HDL (mg/dL)	**0.95**	**0.93-0.97**	**<0.001**	**0.96**	**0.93-0.99**	**0.036**
Total cholesterol (mg/dL)	1.00	0.99-1.01	0.30	─	─	─
LDL (mg/dL)	0.99	0.98-1.01	0.275	─	─	─
Platelet (×10^3^/µL)	1.00	0.99-1.00	0.090	─	─	─
Neutrophils (%)	**1.03**	**1.01-1.05**	**0.007**	1.02	0.99-1.05	0.22
Lymphocyte (%)	0.99	0.97-1.01	0.15	─	─	─
Monocytes (%)	1.04	0.99-1.09	0.11	─	─	─
AI-ECG Score	**3.95**	**2.51-6.20**	**<0.001**	**3.12**	**1.88-5.20**	**<0.001**

Hosmer-Lemshow chi-square (df=8) = 8.565, p = 0.383, R^2^ = 0.383.

**Fig 1:**
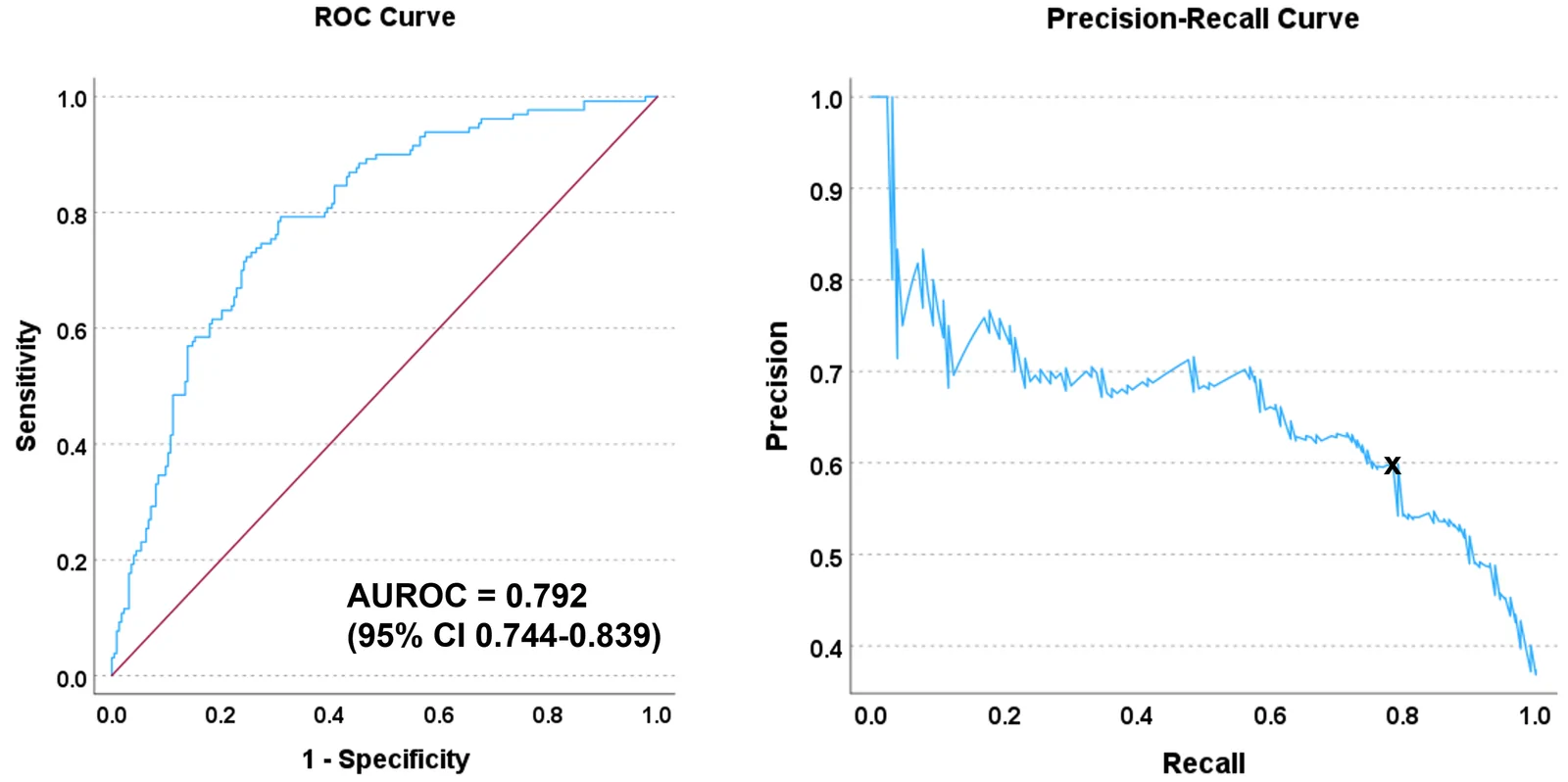
Performance of the adjusted prediction model with the ECG-SMART AI score. This figure shows the receiver operator characteristics (ROC) curve (left) and precision-recall curve (right) of the multivariate logistic regression model using the AI ECG score and other demographic and clinical features. AUROC = area under ROC curve.

Calibration analysis demonstrated a calibration intercept of 0.983 and calibration slope of 1.457. The mean predicted probability of significant coronary artery disease was 26.0%, compared with an observed event rate of 36.1%, indicating that model predictions were generally compressed toward lower probability values in this cohort. Calibration plots are presented in Fig 2.

**Fig 2:**
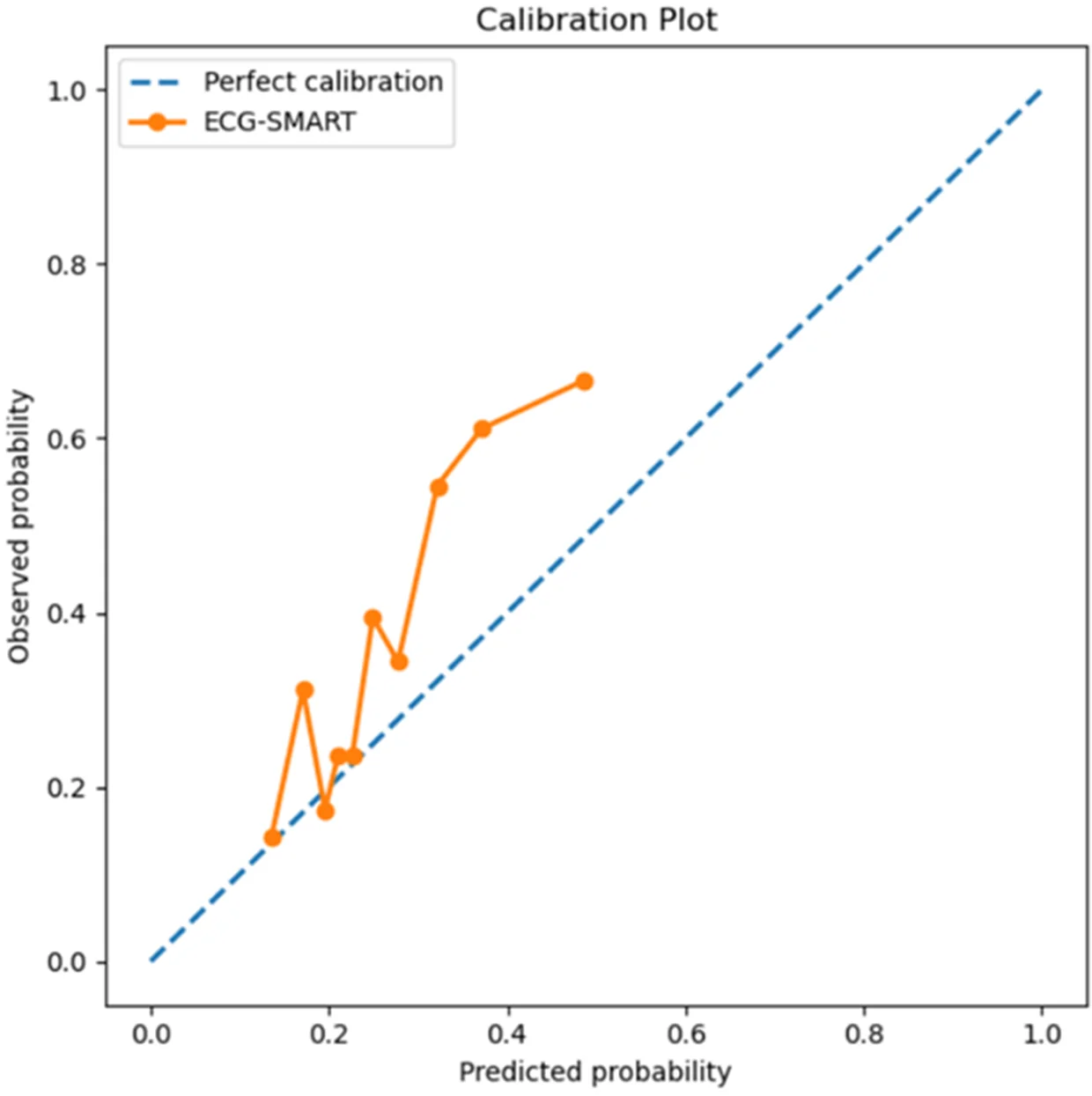
Calibration plot of the ECG-SMART model for predicting significant CAD. Predicted probabilities were grouped into deciles of risk and compared with observed event rates within each decile. The dashed diagonal line represents perfect calibration. Despite good discrimination, this calibration analysis indicates that predicted probabilities were generally lower than the observed prevalence of significant CAD in this cohort. These findings suggest that recalibration may improve risk categorization when the model is deployed in populations with different disease spectra and baseline risk profiles.

In a focused sensitivity analysis stratified by diabetes status, intermediate-to-high AI-ECG risk remained similarly associated with significant coronary artery disease among patients without diabetes (OR 4.0, 95% CI 2.1–7.7) and with diabetes (OR 4.0, 95% CI 2.1–7.8).

### Model interpretability (SHAP analysis)

Fig 3 presents the SHAP beeswarm plot summarizing the most influential ECG features in this cohort. Model predictions were primarily driven by subtle repolarization abnormalities, including global amplitude measurements at the J-point, K-point, and T-peak, as well as regional ST- and T-wave amplitudes in the inferior leads. Additional important features included the Aldrich score in anterior and inferior territories, reflecting estimated myocardial area at risk.

**Fig 3:**
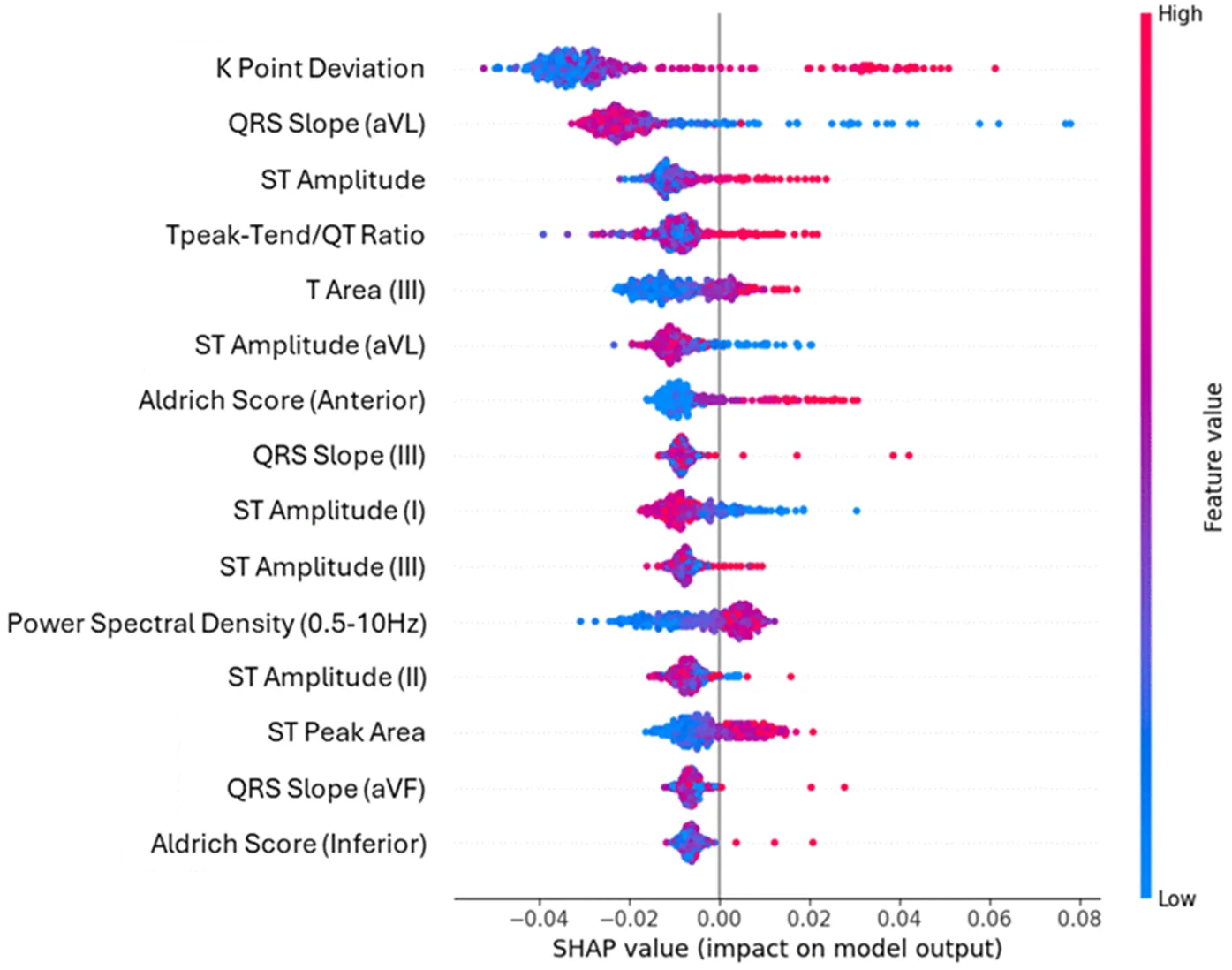
Beeswarm SHAP plot of the most important ECG features. This figure shows the most important ECG features for predicting significant coronary narrowing in the study population. Each dot resembles a patient, and each row (y-axis) resembles an ECG feature. Large SHAP values (x-axis) predict a positive outcome, and negative values predict a negative outcome. Red indicates larger value of that ECG feature.

Markers of repolarization heterogeneity, such as prolonged T-peak–T-end intervals, also contributed to higher predicted risk. Increased low-frequency spectral power further suggested the presence of diffuse myocardial injury currents. Collectively, these findings indicate that the model leveraged distributed, low-amplitude ECG changes rather than overt ischemic patterns to identify patients with significant coronary narrowing. Fig 4 shows two selected examples of correctly identified true positive and true negative cases, further demonstrating the clinical plausibility of ECG features driving these predictions.

**Fig 4:**
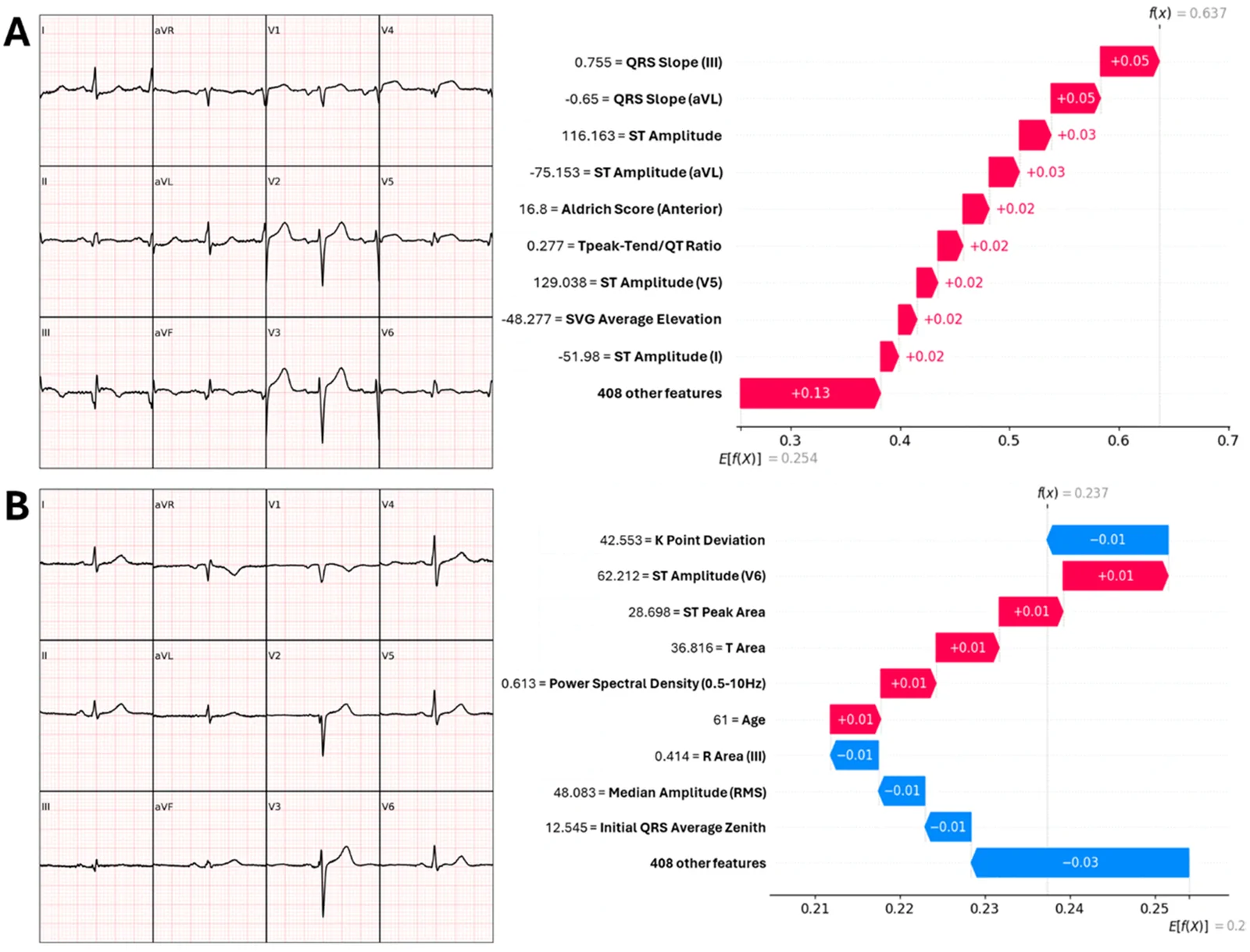
Selected case studies with corresponding waterfall SHAP plots. This figure shows the median beats of the 12-lead ECG and corresponding AI ECG score and waterfall SHAP plots of two case studies: (A) a 55-year-old female patient with past medical history of hypertension and hyperlipidemia scheduled for elective angiography for a 2-week history of chest discomfort and palpitation. The angiogram showed 70% occlusion of the left anterior descending artery. (B) a 61-year-old male patient with a known history of coronary artery disease. An elective angiogram to evaluate chronic chest pain revealed no significant coronary occlusions requiring revascularization.

### Benchmark against a reference standard

To benchmark ECG-SMART against contemporary clinical assessment, we compared its performance with the Risk Factor–Weighted Clinical Likelihood (RF-CL) model recommended by the ESC and AHA guidelines [13]. Because RF-CL is intended for patients undergoing an initial evaluation for suspected coronary artery disease, this analysis was restricted to patients without known coronary artery disease or prior coronary revascularization (n = 199).

Within this subgroup, the adjusted ECG-SMART model demonstrated better discrimination for angiographically significant CAD than the RF-CL model (AUC 0.781 [0.71-0.85] vs. 0.742 [0.67-0.81], S1 Fig). When both predictors were included in the same multivariable logistic regression model, ECG-SMART remained independently associated with significant CAD (P < 0.001), whereas RF-CL was no longer independently associated (P = 0.11). Combining RF-CL with ECG-SMART produced only a modest improvement in discrimination (AUC 0.789).

## Discussion

In this prospective cohort of symptomatic outpatients undergoing elective coronary angiography, we evaluated whether an AI-enhanced ECG model originally developed for acute coronary occlusion could improve estimation of pretest probability for angiographically significant coronary artery disease in a stable clinical setting. The model demonstrated good discrimination (AUROC 0.79) and remained independently associated with angiographically confirmed disease after adjustment for clinical risk factors. Importantly, explainability analyses revealed that predictions were driven by distributed, low-amplitude repolarization abnormalities—such as J-point, K-point, and T-wave features—rather than overt ischemic patterns. These findings support both the biological plausibility and clinical relevance of the model’s predictions. To our knowledge, this is among the first studies to demonstrate the external applicability of an AI-ECG model, originally developed for acute ischemic detection, in identifying anatomically significant CAD in a stable, subacute population.

An additional finding of this study is that the ECG-SMART model compared favorably with the contemporary RF-CL model recommended by clinical practice guidelines for estimating pretest probability of obstructive CAD. Among patients without previously known CAD or prior PCI, the population for which RF-CL was designed, the adjusted ECG-SMART model demonstrated higher discrimination for angiographically significant disease. Furthermore, RF-CL provided little incremental information once ECG-SMART was included in the model, suggesting that AI-derived ECG features capture diagnostic information beyond conventional clinical risk factors and symptom assessment.

Our results extend prior work by showing that ECG-derived AI signatures of acute ischemia may also capture underlying electrophysiologic substrates associated with chronic, obstructive CAD. Although the ECG-SMART model achieved higher performance in its original acute care setting (AUC ~ 0.91 for occlusive myocardial infarction), its performance in this stable cohort remained robust and clinically meaningful. Notably, the model identified patients at increased risk despite the absence of overt ECG abnormalities, addressing a well-recognized limitation of traditional ECG interpretation in subacute settings. This suggests that AI-ECG analysis can augment conventional risk stratification by detecting subtle electrical manifestations of myocardial stress and impaired perfusion, thereby improving estimation of pretest probability for significant CAD prior to invasive evaluation.

An important observation was the difference in calibration between the present cohort and the populations used during model development. The concentration of predictions within the intermediate-risk range likely reflects calibration drift associated with applying a model trained in an acute coronary occlusion population to a lower-risk elective angiography cohort. Although discrimination remained good, calibration analysis demonstrated that predicted probabilities were shifted toward lower values relative to the observed prevalence of significant CAD. This resulted in only a small number of patients being assigned to the highest-risk category despite preserved separation between patients with and without angiographically significant disease. Such calibration shifts are expected when prediction models are transported across populations with different disease spectra and baseline risk profiles. In the present study, nearly half of participants had undergone prior coronary revascularization, reflecting a highly selected referral population with substantial underlying cardiovascular disease burden. These findings suggest that while the electrophysiologic signal captured by the model generalizes beyond acute coronary occlusion, cohort-specific recalibration or threshold adjustment may improve risk categorization when deployed in stable outpatient settings.

Several comorbid conditions can influence ventricular repolarization and ECG morphology independent of CAD, including diabetes-related autonomic dysfunction, prior myocardial injury, structural heart disease, electrolyte abnormalities, and medication effects. However, the ECG-SMART model integrates 417 features spanning multiple electrophysiologic domains and therefore relies on a multidimensional representation of the ECG rather than isolated repolarization markers. Consistent with this, the association between AI-ECG risk and significant CAD was similar among patients with and without diabetes mellitus. Nevertheless, specific measures of autonomic or diabetic neuropathy were not available and future studies should further evaluate their potential influence on AI-ECG predictions.

Recently, significant work has focused on the integration of ML to ECG for the detection of acute cardiac conditions and ACS. This work has been focused on detecting severe CAD in individuals whose ECG is not consistent with occlusive myocardial infarction and arranged for elective coronary angiography. In this cohort study, the AUC of 0.792 aligns with recent findings by Trivedi et al., who demonstrated that deep learning on ECG waveforms can effectively stratify the risk of obstructive stable CAD, even when visual signs of ischemia are absent [8]. This consensus supports the integration of 12-lead ECG with ML, which can capture progressive stable atherosclerosis. This is also supported by Cho et al. (2020), who demonstrated that AI algorithms can significantly enhance the detection of MI compared to standard clinical interpretation, even when utilizing simplified ECG configurations [4].

Although the ECG-SMART model was originally developed to detect acute coronary occlusion, its ability to identify angiographically significant CAD in this study suggests that AI-enhanced ECG analysis captures electrical signatures that extend beyond acute ischemia alone. Whether development of a dedicated AI-ECG model specifically optimized for stable CAD would provide additional diagnostic benefit warrants investigation in future studies.

Unlike in ACS, where there are known ECG features that are being used for intervention, the present study suggests that additional ECG features that are not detected by clinicians represent pathological changes. Such morphological ECG features used by the ECG-SMART model could effectively identify high-risk patients beyond routinely available clinical characteristics. However, rather than replacing established clinical assessment, the ECG-SMART AI score should be viewed as providing complementary diagnostic information beyond routinely available clinical data. The persistent independent association observed after multivariable adjustment suggests that AI-derived ECG features capture electrophysiologic information not fully reflected by conventional cardiovascular risk factors alone. We observed similar results in our prior work,^13^ which established that feature-based ML models provide incremental prognostic value for risk stratification compared to classical clinical scores like HEART or TIMI [14]. By identifying patients with high anatomical risk before they undergo invasive procedures, the AI score could serve as a non-invasive electrical biomarker to optimize the allocation of cardiac angiogram resources.

A study by Tang et al. developed a deep learning-based model to detect CAD using computed tomography angiography as a reference standard for the presence of at least 50% stenosis, and the model achieved an AUC of 0.75 [15]. However, Tang et al. noted that the model struggles to extract valuable features from single-lead data. While traditional pre-test probability models often rely on fixed patient characteristics, this model influences dynamic temporal-spatial ECG features to detect developed pathophysiology changes that may occur.

Our findings suggest that the ECG-SMART model can effectively stratify symptomatic patients into low- and high-risk categories, providing additional noninvasive diagnostic information that may complement existing clinical assessment [5]. However, because this study was conducted in a highly selected cohort of consecutive patients already referred for elective coronary angiography, including a substantial proportion with established CAD and prior coronary revascularization, it was not designed to determine the role of AI-ECG in guiding referral decisions for invasive testing. Rather, this study demonstrates that an AI-ECG model originally developed for acute coronary occlusion generalizes to identifying angiographically significant CAD in an elective evaluation setting. Future prospective studies in broader outpatient populations are needed to determine whether incorporation of AI-ECG into pretest probability assessment can improve diagnostic efficiency, optimize referral pathways, and enhance resource utilization.

### Clinical implications

Our findings suggest that the ECG-SMART AI score may have value beyond its original application in acute coronary occlusion by improving estimation of pretest probability for angiographically significant coronary artery disease among symptomatic patients undergoing elective evaluation. In this setting, AI-ECG risk estimates may complement existing clinical assessment by providing an additional noninvasive source of diagnostic information to help prioritize further diagnostic testing or elective coronary angiography. The present study was not designed to evaluate the model for guiding revascularization decisions or predicting future cardiovascular events.

Consistent with these findings, the greatest potential value of the ECG-SMART model in this population lies in improving diagnostic risk assessment before invasive evaluation rather than informing decisions regarding elective coronary angiography. Whether incorporation of AI-ECG into clinical pathways improves diagnostic efficiency, resource utilization, or patient outcomes remains to be determined in prospective implementation studies.

Importantly, these findings should not be interpreted as supporting routine invasive management of stable CAD. Large randomized clinical trials have demonstrated that an initial invasive strategy does not reduce death or myocardial infarction compared with optimal medical therapy in appropriately selected patients with stable ischemic heart disease [16,17]. Rather, the potential value of AI-enhanced ECG analysis lies in improving estimation of pretest probability for anatomically significant CAD and complementing existing clinical assessment by identifying patients who may benefit from further diagnostic evaluation.

From an implementation perspective, the ECG-SMART framework is device-agnostic and can be integrated into existing ECG workflows without additional burden on clinicians. The use of interpretable outputs further supports clinical adoption by aligning model predictions with physiologically meaningful ECG features. It is worth noting that, though, the tool can probably be restricted to prioritizing who gets scheduled for angiogram first and who can wait, rather than identifying true negative patients who do not need any further evaluation at all. Prospective validation and integration into clinical decision pathways will be important next steps to determine its impact on diagnostic efficiency, resource utilization, and patient outcomes.

### Study limitation

This study has several limitations. First, the probability thresholds used for risk categorization were originally derived from cohorts evaluated for acute coronary occlusion and were not specifically optimized for stable outpatient populations undergoing elective angiography. Although discrimination remained robust, calibration analyses suggested that threshold recalibration may improve probability estimation and risk categorization in populations with different disease prevalence and clinical characteristics. Second, the study was conducted at a single tertiary care center, which may limit generalizability to other practice settings and patient populations. Additional validation across more diverse populations, geographic regions, and ECG acquisition systems is needed to confirm robustness and device-level generalizability. Third, although the cohort included sufficient outcome events for the primary ROC and multivariable logistic regression analyses, the single-center sample size limits precision for subgroup and exploratory analyses. Fourth, the study population consisted of symptomatic patients referred for elective angiography, introducing potential referral bias and limiting applicability to asymptomatic or lower-risk populations. Finally, while angiographic stenosis was used as the reference standard, it does not fully capture functional ischemia; future studies incorporating physiologic assessments (e.g., fractional flow reserve) may provide additional insights.

## Conclusion

In this prospective cohort of symptomatic outpatients, the ECG-SMART AI-ECG score demonstrated good discrimination for detecting significant coronary artery disease and remained independently associated with angiographic findings after adjustment for clinical risk factors. These results support its role as a noninvasive electrical biomarker that generalizes beyond acute care settings to patients undergoing elective evaluation.

Integration of AI-ECG–based risk estimates into routine clinical assessment in outpatient settings may improve estimation of pretest probability for anatomically significant coronary artery disease and complement existing strategies used to prioritize elective diagnostic evaluation. Prospective studies are needed to determine whether incorporation of AI-ECG into clinical decision pathways improves diagnostic efficiency, resource utilization, and patient-centered outcomes.

## Supporting information

S1 Fig Performance of ECG-SMART AI score against conventional clinical risk estimation in patients with no known history of CAD. Receiver operating characteristic curves comparing the ECG-SMART AI score and RF-CL model in patients without known coronary artery disease.
(TIF)

S1 Data Data. (XLSX)
